# Use of Continuous Etomidate Infusion to Rapidly Correct Hypercortisolism in a Patient With Disseminated Nocardiosis

**DOI:** 10.7759/cureus.20214

**Published:** 2021-12-06

**Authors:** Azka Tasleem, Melissa Cavaghan, Quinn A Czosnowski, Zeb Saeed

**Affiliations:** 1 Internal Medicine, Indiana University Health Ball Memorial Hospital, Muncie, USA; 2 Endocrinology and Diabetes, Indiana University Health, Indianapolis, USA; 3 Pharmacy, Indiana University Health, Indianapolis, USA

**Keywords:** cavitary lung lesions, hypercortisolism, cushing's syndrome, etomidate, disseminated nocardiosis

## Abstract

Cushing’s syndrome (CS) is an immunocompromised state characterized by impaired cellular and adaptive immunity due to hypercortisolism. This imbalance in the immune system leads to a high risk of opportunistic infections which can potentially prove fatal. In such patients, mortality can be reduced with early diagnosis and effective management of the underlying hypercortisolism. In this case report, we describe how prompt reduction of cortisol levels using a low dose continuous etomidate infusion was pivotal in effective treatment of an opportunistic infection, disseminated nocardiosis, in a 29-year-old female with Cushing’s syndrome. We also discuss how treatment with antibiotics including empiric therapy with Imipenem and sulfamethoxazole/trimethoprim (SMX/TMP) and definite therapy as per susceptibility testing, with amikacin, SMX/TMP, and doxycycline helped to prevent adverse outcomes. Through this case, we aim to emphasize that infiltrates or cavitary lesions on the computed tomography (CT) scan of the chest in a patient with Cushing’s syndrome should raise concern for nocardiosis, and prompt management with antibiotics should be initiated. Similarly, disseminated nocardiosis should always raise concern for possible immune deficiency states like Cushing’s syndrome. Our case is unique in detailing the significance of using etomidate to acutely lower cortisol levels in a patient with endogenous CS and widespread invasive opportunistic infection. The pharmacology aspects of the Etomidate, in this case, have been published in the Journal of Pharmacy Practice and cited appropriately in this article.

## Introduction

Cushing's syndrome (CS) is a disease characterized by prolonged hypercortisolism. Endogenous Cushing's syndrome is associated with increased morbidity and mortality, and prompt diagnosis and treatment are essential in mitigating the detrimental effects of cortisol excess. Infections occurring due to impaired cellular immunity from excess circulating cortisol are among the leading cause of death in CS [1]. In such cases, resolution of underlying hypercortisolism is pivotal in treating the infection. While resection of the pathogenic tumor remains the mainstay treatment of CS, medical therapy is instituted in pre- and peri-operative settings as a bridge to surgery, when surgery is not feasible, in acute, potentially life-threatening circumstances, or cases of persistence or recurrence after surgery [2].

Nocardia is a filamentous Gram-positive bacterium that can cause invasive and disseminated infections in immunocompromised hosts. A previously healthy patient with invasive nocardiosis should be investigated for the presence of cellular immunodeficiency given this strong association. Invasive nocardiosis most frequently presents with pulmonary disease, followed by disseminated disease, which is defined as the involvement of at least two noncontiguous organs and/or demonstration of bloodstream infection. Disseminated nocardiosis, which affects 12-50% of patients with nocardial infection is associated with mortality rates of 34.5-40% [3]. The immunocompromised states associated with disseminated nocardiosis are organ transplant recipients, long-term exogenous corticosteroid use, malignancies, and a history of HIV infection [4]. While studies have described the occurrence of invasive nocardiosis in endogenous CS, the association may be overlooked by internists given that endogenous CS itself is an uncommon diagnosis. Hence, we present a case of a young woman who presented with life-threatening disseminated nocardiosis in the setting of severe endogenous hypercortisolism, where timely use of etomidate was pivotal in management.

## Case presentation

**Figure 1:**
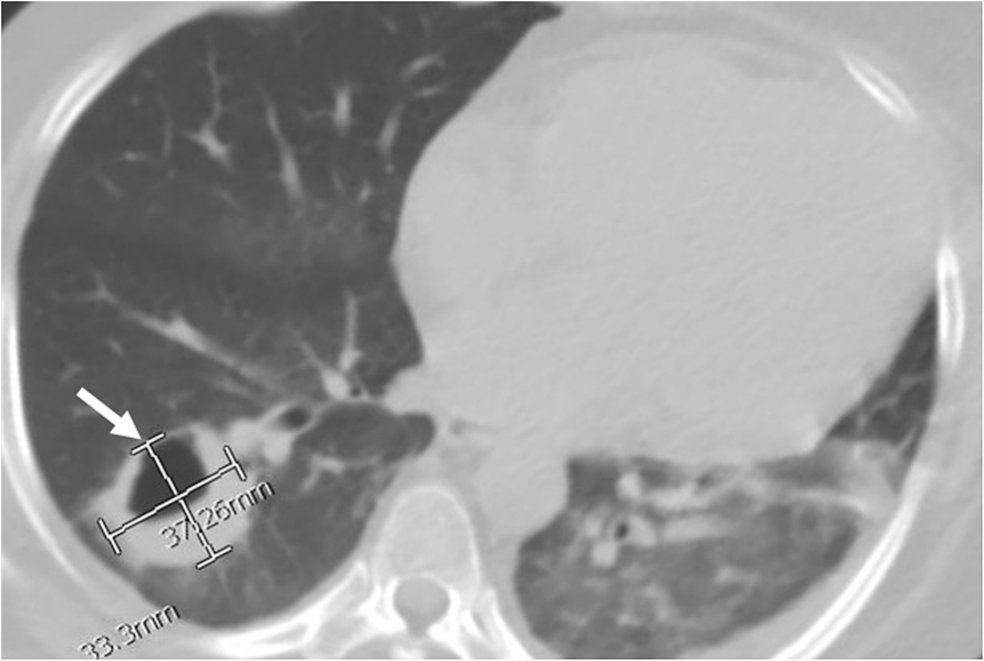
Transverse view of CT scan of chest without contrast showing a right lung cavitary lesion with thickened walls measuring 3.3 cm x 3.7 cm.

A 29-year-old African American female with a history of hypertension, untreated Cushing’s syndrome, and type 2 diabetes mellitus was transferred from an outside facility with disseminated pulmonary and intracranial nocardiosis. She had initially presented with left lower quadrant abdominal pain and was found to have a urinary tract infection with *Escherichia coli*. The physical examination was pertinent for central adiposity, hirsutism, the presence of dorsal cervical and supraclavicular fat pads, and wide purple striae on the abdomen and thighs. On the second day of admission, she developed acute respiratory failure requiring 4 L of oxygen via nasal cannula. A CT chest revealed a 4.8 cm thick-walled right lower lobe cavitary mass and multiple nodules in the lungs bilaterally (Figure 1).

**Figure 2:**
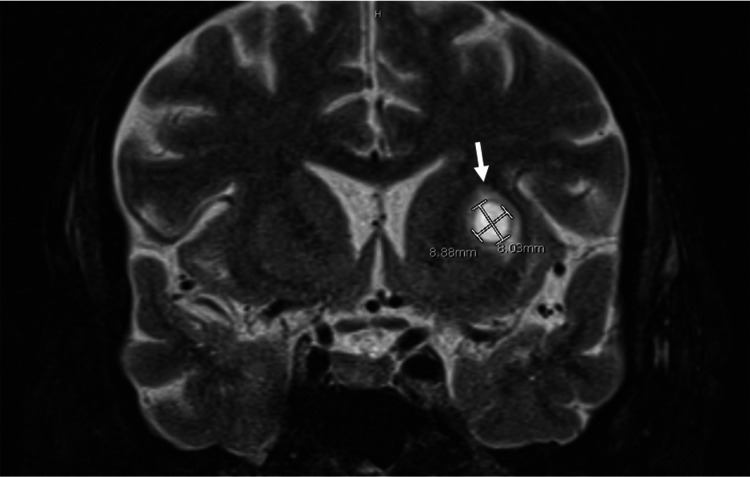
MRI scan of the brain, coronal section, showing lesion in the left anterior lentiform nucleus measuring 8.8 mm x 8 mm with surrounding edema.

**Figure 3:**
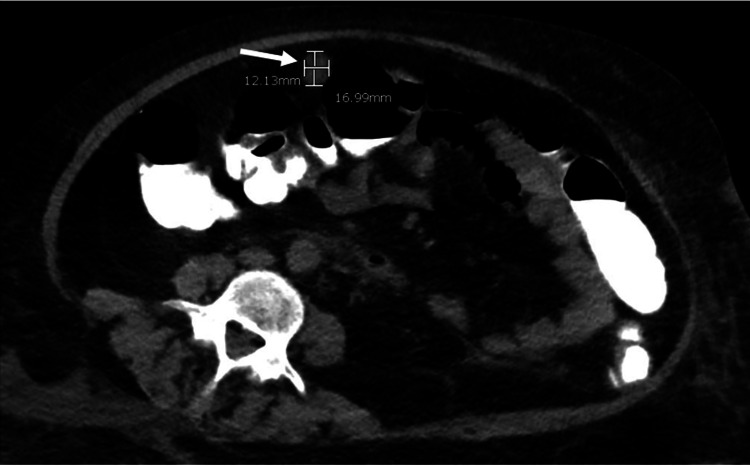
Transverse view of CT scan of the abdomen without contrast showing a peritoneal nodule measuring 1.2 cm x 1.7 cm.

Subsequently, bronchial lavage revealed branching Gram-positive bacilli that stained positive with modified acid-fast stain, suspicious for Nocardia. CT-guided biopsy of the right cavitary mass also revealed the presence of a filamentous branching organism with the culture-positive for *Nocardia farcinica*. Brain MRI with gadolinium contrast and CT abdomen without contrast further showed multiple cerebral and cerebellar peripherally enhancing lesions, the largest being 10 mm in size, and peritoneal fat nodules, respectively, confirming the diagnosis of disseminated nocardiosis (Figures 2, 3).

In the outside facility, the patient was treated with IV trimethoprim/sulfamethoxazole (TMP/SMX) 480 mg every eight hours and IV imipenem 1 g every eight hours. Oral linezolid 600 mg every 12 hours was also added for the treatment of disseminated nocardiosis which was discontinued due to thrombocytopenia before transfer to our facility. No steroids were given.

Upon transfer to our facility, treatment was continued with IV imipenem 1 g IV every six hours and IV sulfamethoxazole/trimethoprim 480 mg every eight hours. Blood cultures were negative for Nocardia and any other microorganisms. The various tests done during the course of hospital stay are tabulated below (Table 1).

**Table 1:** Various tests conducted to reach the diagnosis. ACTH: adrenocorticotropic hormone; CRH: corticotropin-releasing hormone

Investigation	Result
Cryptococcal antigen	Negative
Histoplasma antigen	Negative
Tspot	Negative
Human immunodeficiency virus (HIV)	Negative
Toxoplasma antibody	Negative
At the time of diagnosis (1 year back) 24-hr urine free cortisol (normal range; 3.1-42.3)	1913 mcg
At the time of diagnosis (1 year back), serum cortisol (normal range: 4-25)	34.8 mCg/dL
At this hospital stay, random serum cortisol range (normal range: 4-25)	43.9-63.3 mCg/dL
At this hospital stay, ACTH range (normal range: 6-50)	197-378 pg/mL
CRH stimulation test	ACTH rise from 318 to 992 pg/mL suggestive but not diagnostic of a pituitary etiology.
8-mg dexamethasone suppression test	Partial but not complete suppression of cortisol, the levels of cortisol dropped from 44 to 11 mg/dL (but not <1.8 mg/dL)

Since corticotropin-releasing hormone (CRH) stimulation test and 8 mg dexamethasone suppression test were inconclusive, inferior petrosal sinus sampling (IPSS) was planned to determine the source of adrenocorticotropic hormone (ACTH). However, within 48 hours of admission, the patient had worsening respiratory status necessitating intensive care (ICU). Thus, further evaluation with IPSS was deferred, and the patient was started on etomidate infusion to rapidly control the hypercortisolemia. Etomidate infusion resulted in normalization of cortisol levels within 24 hours and was continued for five days, after which treatment was continued on oral ketoconazole 400 mg three times a day and metyrapone 250 mg four times a day with continued normalization of serum cortisol. She was subsequently successfully transferred out of the ICU on day six of hospitalization.

The final results of the bronchial lavage cultures came back as multi-drug-resistant *Nocardia farcinica* which was resistant to amoxicillin-clavulanate and imipenem and sensitive to amikacin, ceftriaxone, doxycycline, and TMP/SMX. The antibiogram is shown below (Table 2).

**Table 2:** Antibiogram showing sensitivity of Nocardia farcinica to various antibiotics. S: sensitive; R: resistant: I: intermediate

Antibiotic	MIC	Interpretation
Amikacin	<=1	S
Amoxicillin clavulanate	64/32	R
Ceftriaxone	8	S
Ciprofloxacin	>4	R
Clarithromycin	>16	R
Doxycycline	1	S
Imipenem	16	R
Linezolid	2	S
Minocycline	2	I
Moxifloxacin	4	R
Tobramycin	<=1	S
Trimethoprim/Sulfa	1/19	S

Based on the sensitivities, our patient was switched from imipenem to IV amikacin 250 mg every 48 hours, oral doxycycline 100 mg every 12 hours, and SMX/TMP 800-160 mg two tablets every six hours with a plan to continue IV amikacin for a total of six weeks and oral therapy with doxycycline and SMX/TMP for 12 months in total.

Repeat cross-sectional imaging done two weeks after initiation of therapy showed stable pulmonary nodules and a decrease in the peritoneal lesions. As the patient clinically improved, ketoconazole and metyrapone were held for seven days to allow the cortisol to rise and facilitate a diagnostic IPSS. IPSS results showed that the central-to-peripheral baseline ACTH gradient was 8.7 (>2 indicative of pituitary source of ACTH). Also, central-to-peripheral post-CRH ACTH gradient was 25 (>3 indicative of pituitary source of ACTH). Hence, the results confirmed the pituitary gland as the source of ACTH, establishing the diagnosis of Cushing's disease. Also, MRI of the sella was notable for a 2.4 mm pituitary adenoma, while the CT abdomen did not exhibit any adrenal pathology. Neurosurgery was consulted, and the patient underwent trans-sphenoidal hypophysectomy on day 39 of the hospital stay. Post-operatively, the patient was started on replacement doses of hydrocortisone 20 mg in the morning and 10 mg at night, suggesting successful surgical intervention and cure.

Our patient completed six weeks of intravenous amikacin therapy. A few days before discharge, she developed a facial rash attributed to TMP-SMX, which was then switched to IV ceftriaxone 1 g every 24 hours. The patient continued to improve and was finally discharged to a rehabilitation facility on day 63 of hospitalization on doxycycline 100 mg every 12 hours and ceftriaxone 1 g every 24 hours.

## Discussion

Excess glucocorticoids, exogenous and endogenous, have long been known to alter the host response to infectious processes. Our case not only highlights the importance of recognizing endogenous Cushing’s syndrome (CS) as an important differential for the immunocompromised state leading to opportunistic infections but also the importance of correcting the hypercortisolism rapidly to allow for effective treatment of the infection.

Glucocorticoids exert many complex quantitative and qualitative immunosuppressive effects both to the circulating neutrophils and lymphocytes. There is decreased migration of neutrophils to areas of inflammation and reduced phagocytic and bactericidal activity. Elevated steroid levels also lead to lymphopenia, presumably by increased lysis of lymphocytes and abnormal lymphocyte redistribution [5]. This greatly hampers cellular and adaptive immunity, which predisposes individuals to organisms that would not invade an immune-competent host. It is no surprise that infections are among the leading cause of death in patients with CS and the risk of infections in patients with elevated cortisol levels remains markedly elevated for at least a year even after treatment of hypercortisolism [6,7]. Moreover, severe CS is defined by urine-free cortisol at least five times the upper limit of normal. In such patients, prophylaxis with SMX/TMP is recommended [8].

A dose-response relationship is seen with the type and severity of opportunistic infections and CS with studies showing that patients with cortisol levels >43.1 mcg/dL and urine free cortisol (UFC) >2000 mcg are at the highest risk of severe infections. This parallels our patient who had serum cortisol values in the range of 43.9-63.3 mCg/dL and UFC 1910 mcg. In a review of CS and nocardiosis, all cases of nocardiosis in patients described in the literature with CS have been ACTH dependent, with a recent review observing that over 80% were caused by ectopic CS [9]. Moreover, the ACTH value described in such cases is very elevated with the average being 441.8 ± 131.8 pg/mL, in tandem with our case (378 pg/mL), signifying the severity of hypercortisolism. Our case is the third case of pituitary Cushing’s (Cushing's disease) being the etiology of hypercortisolism in a patient with invasive nocardiosis described in the medical literature.

Treatment of disseminated nocardiosis is complicated by variable antibiotic resistance. Therefore, an initial antibiotic regimen typically contains two to three drugs with one being TMP/SMX. Subsequent alterations of antimicrobial therapies should be based on organism sensitivities and response to therapy [10]. Details of the etomidate infusion have been published elsewhere [10]. Despite that, the mortality of nocardiosis is very high at around 40% with the mortality of this infection in CS being reported to be even higher at 61-66% [11]. The likely reason for higher mortality in patients with CS is due, in part, to the unmitigated high circulating glucocorticoid levels and the lag that occurs between the diagnosis of CS and resection of the primary ACTH-producing tumor. Cases of ectopic CS can be diagnostic challenges, with the hormonal source difficult to find. Even when the source is identified, many of these patients are too sick to undergo any surgical interventions. Medical therapy as a bridge to surgical intervention can be life-saving in such cases. Several drugs are now commercially available for use in CS, including steroidogenesis inhibitors (ketoconazole, metyrapone, mitotane, and etomidate), centrally-acting agents (somatostatin analogs), and glucocorticoid receptor antagonists (mifepristone) [12]. Our case is the only case in the existing literature that describes using etomidate acutely to lower serum cortisol levels in a patient with disseminated nocardiosis.

Initially introduced as a sedative in the 1970s, etomidate blocks multiple enzymes in the steroidogenesis pathway, including the 11-beta-hydroxylase, 21-hydroxylase, and side-chain cleavage enzyme, thus lowering the synthesis of cortisol [13]. Compared to other medications for Cushing syndrome, etomidate offers the benefit of a more rapid onset of action of a few minutes. In emergent cases like ours where prompt management of hypercortisolemia is necessary to prevent adverse outcomes, etomidate offers a unique benefit with its quick on, quick off action. Moreover, nearly all the other medical therapies of CS are oral agents and often such patients are too sick to take oral drugs. Several protocols for etomidate infusions in CS exist with rates ranging from 0.03 to 0.3 mg/kg/h been found effective [14]. While higher rates result in quicker normalization of serum cortisol, there is a higher risk of sedation/hypnosis at such doses. On the contrary, lower doses at 0.02 mg/kg/h have a lower frequency of side effects but may result in a slower resolution in serum cortisol than desired. Thus, in our patient, we gave an initial dose of 0.3 mg/kg followed by an infusion, initially started at a rate of 0.1 mg/kg/h and titrated to serum cortisol levels which resulted in resolution of hypercortisolism within 24 hours with no untoward side effects to our patient. Initially, cortisol levels were checked after two hours but as levels improved, they were monitored every four to six hours. Etomidate served as an effective bridge to other medical therapies and eventual surgery in our case while maintaining a state of normal cortisol levels which likely was pivotal in the treatment of disseminated nocardiosis.

Our case is the first case described in the literature of effectively using etomidate infusion in rapidly correcting hypercortisolemia in a patient with disseminated nocardiosis with underlying CS. We hope this encourages clinicians not only to consider endogenous CS as a possible etiology of disseminated nocardiosis but also to attempt to promptly normalize circulating glucocorticoid levels to allow for effective treatment.

## Conclusions

Invasive nocardiosis should always be investigated in a patient presenting with signs of infection in the presence of immunodeficiency such as Cushing’s syndrome. Treatment of hypercortisolism is needed in effective therapy of such opportunistic infections. Etomidate is the drug of choice considering its benefits over other drugs since it allows for rapid reduction of elevated serum cortisol levels and can be used safely and effectively in patients with Cushing’s disease and widespread life-threatening infections.

## References

[1] Preda VA, Sen J, Karavitaki N, Grossman AB (2012). Etomidate in the management of hypercortisolaemia in Cushing's syndrome: a review. Eur J Endocrinol.

[2] Heyn J, Geiger C, Hinske CL, Briegel J, Weis F (2012). Medical suppression of hypercortisolemia in Cushing's syndrome with particular consideration of etomidate. Pituitary.

[3] Williams E, Jenney AW, Spelman DW (2020). Nocardia bacteremia: a single-center retrospective review and a systematic review of the literature. Int J Infect Dis.

[4] Rawat D, Rajasurya V, Chakraborty RK, Sharma S (2021). Nocardiosis. StatPearls [Internet].

[5] Fauci AS, Dale DC, Balow JE (1976). Glucocorticosteroid therapy: mechanisms of action and clinical considerations. Ann Intern Med.

[6] Dekkers OM, Horváth-Puhó E, Jørgensen JO (2013). Multisystem morbidity and mortality in Cushing's syndrome: a cohort study. J Clin Endocrinol Metab.

[7] Valassi E, Tabarin A, Brue T (2019). High mortality within 90 days of diagnosis in patients with Cushing’s syndrome: results from the ERCUSYN registry. Eur J Endocrinol.

[8] Nieman LK, Biller BM, Findling JW, Murad MH, Newell-Price J, Savage MO, Tabarin A (2015). Treatment of Cushing's syndrome: an endocrine society clinical practice guideline. J Clin Endocrinol Metab.

[9] Zhang D, Jiang Y, Lu L, Lu Z, Xia W, Xing X, Fan H (2021). Cushing's syndrome with nocardiosis: a case report and a systematic review of the literature. Front Endocrinol (Lausanne).

[10] Hays WB, Czosnowski Q (2021). Continuous etomidate for the management of Cushing's syndrome complicated by pulmonary nocardiosis. [In Press]. J Pharm Pract.

[11] Xu L, Xu Q, Yang M, Gao H, Xu M, Ma W (2016). Nocardiosis in ectopic ACTH syndrome: a case report and review of 11 cases from the literature. Exp Ther Med.

[12] Daniel E, Newell-Price JD (2015). Therapy of endocrine disease: steroidogenesis enzyme inhibitors in Cushing's syndrome. Eur J Endocrinol.

[13] Johnson TN, Canada TW (2007). Etomidate use for Cushing's syndrome caused by an ectopic adrenocorticotropic hormone-producing tumor. Ann Pharmacother.

[14] Schulte HM, Benker G, Reinwein D, Sippell WG, Allolio B (1990). Infusion of low dose etomidate: correction of hypercortisolemia in patients with Cushing's syndrome and dose-response relationship in normal subjects. J Clin Endocrinol Metab.

